# Compatibility systems and pollinator dependency in morning glory species (Convolvulaceae)

**DOI:** 10.1186/s12870-023-04437-y

**Published:** 2023-09-15

**Authors:** Piriya Hassa, Paweena Traiperm, Alyssa B. Stewart

**Affiliations:** Department of Plant Science, Faculty of Science, Mahidol University, Bangkok, 10400 Thailand

**Keywords:** Bees, Biodiversity, Breeding systems, Pollination ecology, Pollinator dependence patterns, Self-compatibility, Terrestrial

## Abstract

**Background:**

The Convolvulaceae is a large family containing species exhibiting a range of breeding systems and pollinated by diverse animal taxa. We studied the pollination ecology of 15 Convolvulaceae species, representing seven genera (*Argyreia* Lour., *Camonea* Raf., *Evolvulus* L., *Hewittia* Wight & Arn., *Ipomoea* L., *Merremia* Dennst. ex Endl., and *Operculina* Silva Manso), in northeastern Thailand, a family that is highly diverse yet understudied in the paleotropics. Specifically, we studied their compatibility systems and degrees of pollinator dependency using pollination experiments, examined pollinator composition and visitation rates using video observation, and determined if there is an association between pollinator visitation rates and degree of pollinator dependence.

**Results:**

Our results showed that most species are self-compatible, but the degree of pollinator dependence varies. Six species were found to be highly dependent on pollinators, as two are self-incompatible and four are self-compatible but had reduced seed set when pollinators were excluded, possibly due to herkogamy. Seven species showed low dependence on pollinators and seed set remained high when pollinators were excluded. Pollinator dependence was inconclusive for two species as seed set was low in all pollination treatments. We also found an association between pollinator visitation rates and degree of pollinator dependence. Specifically, species exhibiting high pollinator dependence received frequent visits from pollinators, while species exhibiting low pollinator dependence either received frequent visits from pollinators (and received high amounts of xenogamous pollen) or infrequent visits from pollinators (and received significantly lower amounts of xenogamous pollen). Most of our study species were primarily visited by bees (e.g., *Lasioglossum*, *Amegilla*, *Apis*, and meliponines), with the exception of one night-blooming species that was visited primarily by crepuscular butterflies and hawkmoths.

**Conclusions:**

The cumulative findings of this study demonstrate how pollinator dependence is influenced by breeding system, and suggest that pollinator visitation is consistently high for species exhibiting high pollinator dependence but varies across species exhibiting low pollinator dependence. Our findings are also important for assessing the conservation risks of paleotropical Convolvulaceae.

**Supplementary Information:**

The online version contains supplementary material available at 10.1186/s12870-023-04437-y.

## Background

The vast majority of angiosperms depend on animals for pollination, particularly in tropical areas where an estimated 94% of species benefit from animal pollinators [1]. Yet many plant species are actually self-compatible, ranging from 41% of species in mainland communities to 66% of species in island communities [2], which allows them to produce offspring even without cross pollination by animals [3]. Animal-pollinated species that are self-incompatible obviously rely on pollinators to transfer pollen across individuals in order to reproduce [4]. However, even self-compatible species can benefit from animal pollinators, as cross pollination promotes gene flow within and between populations, which increases genetic diversity, reduces inbreeding depression, and facilitates adaptation to environmental changes [5]. Therefore, across the immense diversity of flowering plants, not only do we observe variation in breeding systems, we also observe variation in pollinator dependence [6, 7].

The degree of dependence on pollinators by animal-pollinated plant species is determined by numerous factors. Breeding system is an important determinant, as self-incompatible species are inherently more dependent on pollinators than self-compatible species [7]. Moreover, dioecious and monoecious species are generally more dependent on pollinators than hermaphroditic species [5]. Yet even among hermaphroditic species, those exhibiting herkogamy and/or dichogamy may be highly dependent on pollinators [5]. Degree of pollinator dependence can be assessed using pollinator-exclusion experiments [6, 8]. If reproductive output is similar for animal-pollinated flowers and pollinator-excluded flowers, pollinator dependence is low, but if reproductive success is reduced in the pollinator-excluded treatment compared to control (animal-pollinated) flowers, pollinator dependence is high [6, 8].

The advantages of outcrossing are well known [9], yet dependence on animal pollinators also carries some risks. Such risks include reduced reproductive success when pollinators or mates (i.e., other conspecific plants) are rare, unreliable, or completely absent [10], as well as an increased risk of interspecific pollen transfer unless plants have mechanisms for precise pollen placement [11, 12]. Such pollination-uncertain environments can favor the evolution of reproductive assurance mechanisms, in which plant species are able to self-pollinate to ensure reproduction when xenogamous pollen is absent or insufficient [10, 13]. The prevalence of mixed mating systems found in nature [5, 14] is thought to be due in part to the pervasiveness of pollen limitation and selection for reproductive assurance [10, 13, 15]. Thus, plants are continuously under the selective forces of reproductive assurance versus inbreeding depression [16–18], and numerous transitions between self-compatibility and self-incompatibility have occurred [19].

While variation in compatibility systems has been studied extensively [5, 20, 21], variation in pollinator dependence has received less attention (but see [6, 7, 22]). We therefore examined both the compatibility systems and degree of pollinator dependence across convolvulaceous species in Thailand. The Convolvulaceae is a large family with over 1,840 known species (sensu [23]) and exhibiting diverse floral morphologies [23–25], breeding systems [26–28], and pollinators [29–31]. However, most research on the breeding systems and pollinators of the Convolvulaceae has been conducted in the Neotropics [18, 28, 32–34] and temperate zones [35–38], and we still lack information from paleotropical areas (but see [39–41]. Such underrepresentation needs to be remedied, especially given that the center of diversity for many Convolvulaceae genera is located in the Paleotropics (e.g., *Argyreia* Lour., *Erycibe* Roxb., *Stictocardia* Hallier f.) [23].

The objectives of this work were therefore to (1) study the compatibility systems and degree of pollinator dependence in Thai Convolvulaceae using pollination experiments, (2) examine pollinator composition and visitation rates using video observation, and (3) determine if there is an association between pollinator visitation rates and degree of pollinator dependence. Based on compatibility systems and pollinator activity, there are three main patterns of pollinator dependence that can arise. The first pattern is low pollinator dependence with high outcrossing rates, which we would expect from self-compatible species with autonomous self-pollination that receive abundant xenogamous pollen. Because they receive sufficient visits from pollinators, these species may not need to rely on reproductive assurance mechanisms. The second pattern is low pollinator dependence with low outcrossing rates, which we would expect from self-compatible species with autonomous self-pollination that receive little xenogamous pollen. Because they receive insufficient visits from pollinators, these species are expected to rely on reproductive assurance mechanisms. The third pattern is high pollinator dependence, which we would expect from self-incompatible species or from self-compatible species that experience low levels of autonomous self-pollination. For these species, reproductive success is highly dependent on the quantity and quality of pollinator visits. We hypothesized that plant species exhibiting high pollinator dependence would receive more frequent and consistent pollinator visitation than those exhibiting low pollinator dependence. This study aims not only to provide information on the pollination ecology of under-studied paleotropical Convolvulaceae, but to improve our understanding of variation in pollinator dependence across related plant taxa and how it relates to pollinator visitation rates.

## Methods

### Study area

**Fig. 1:**
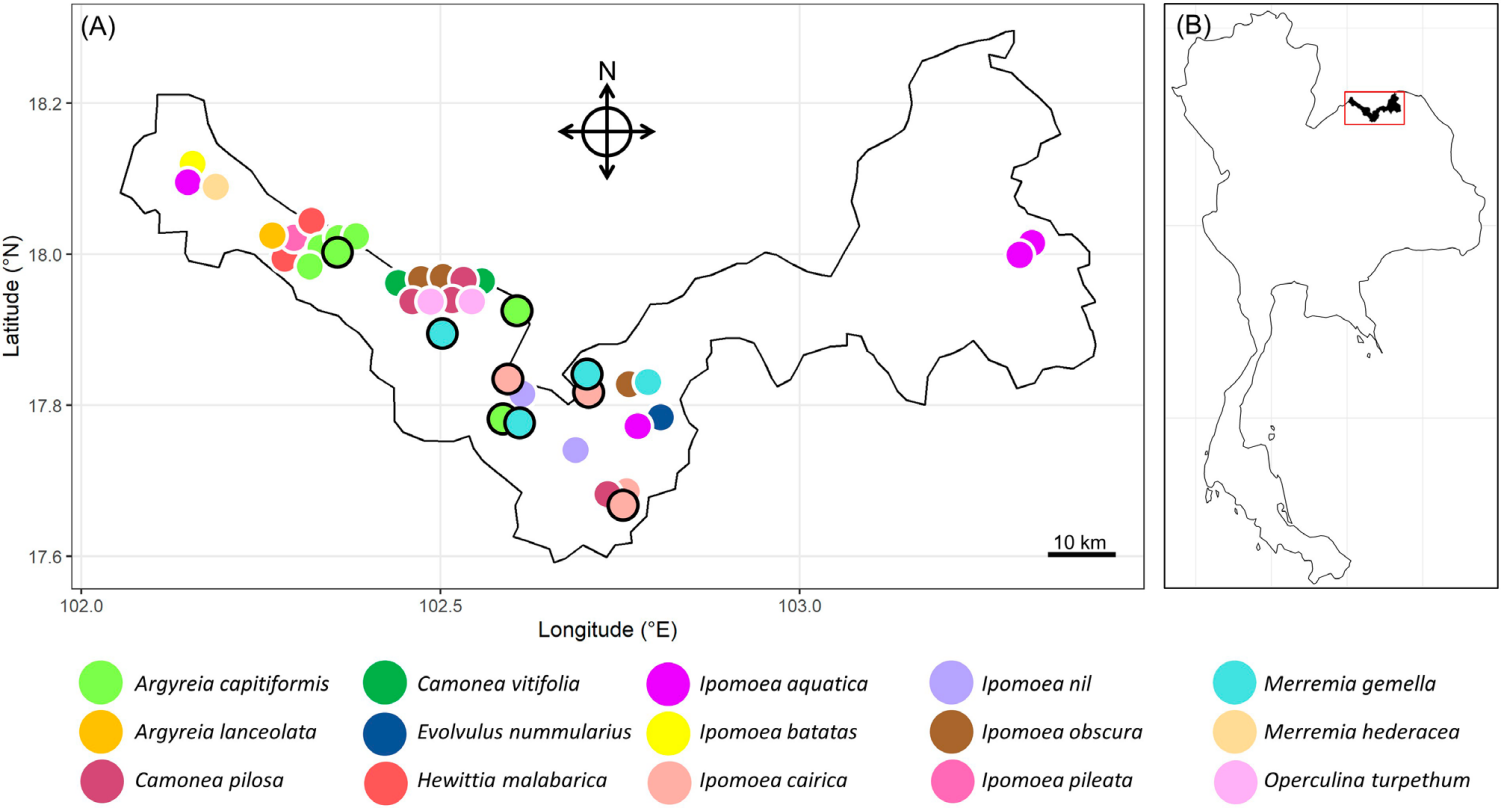
Study area in Nong Khai, Thailand. (**A**) Map of Nong Khai province showing the locations where data were collected for each of the 15 Convolvulaceae study species, represented by different colors. Colored circles with a white outline were examined in 2017–2019, while colored circles with a black outline were examined in 2022–2023. (**B**) Location of Nong Khai province within Thailand. Maps were created in R using packages “ggmap” and “ggplot2”

This study was conducted in Nong Khai province in northeastern Thailand (Fig. 1), where numerous Convolvulaceae species are found [23]. These plants typically grow in sunny and moist areas [23]. Common habitats where we observed Convolvulaceae include near rice paddy fields, along rural roads, and in mixed deciduous forests. We conducted fieldwork between December 2017 to March 2019, with additional pollination experiments conducted during December 2022 – January 2023 for three species that had inconclusive results from our first round of experiments (see “Pollination experiments” section below). The average yearly precipitation in Nong Khai was 1,766.7 mm (years 2017–2019) [42]. The average yearly temperature ranged between 26-30^o^C (years 2017–2019) [42].

### Study species

**Fig. 2:**
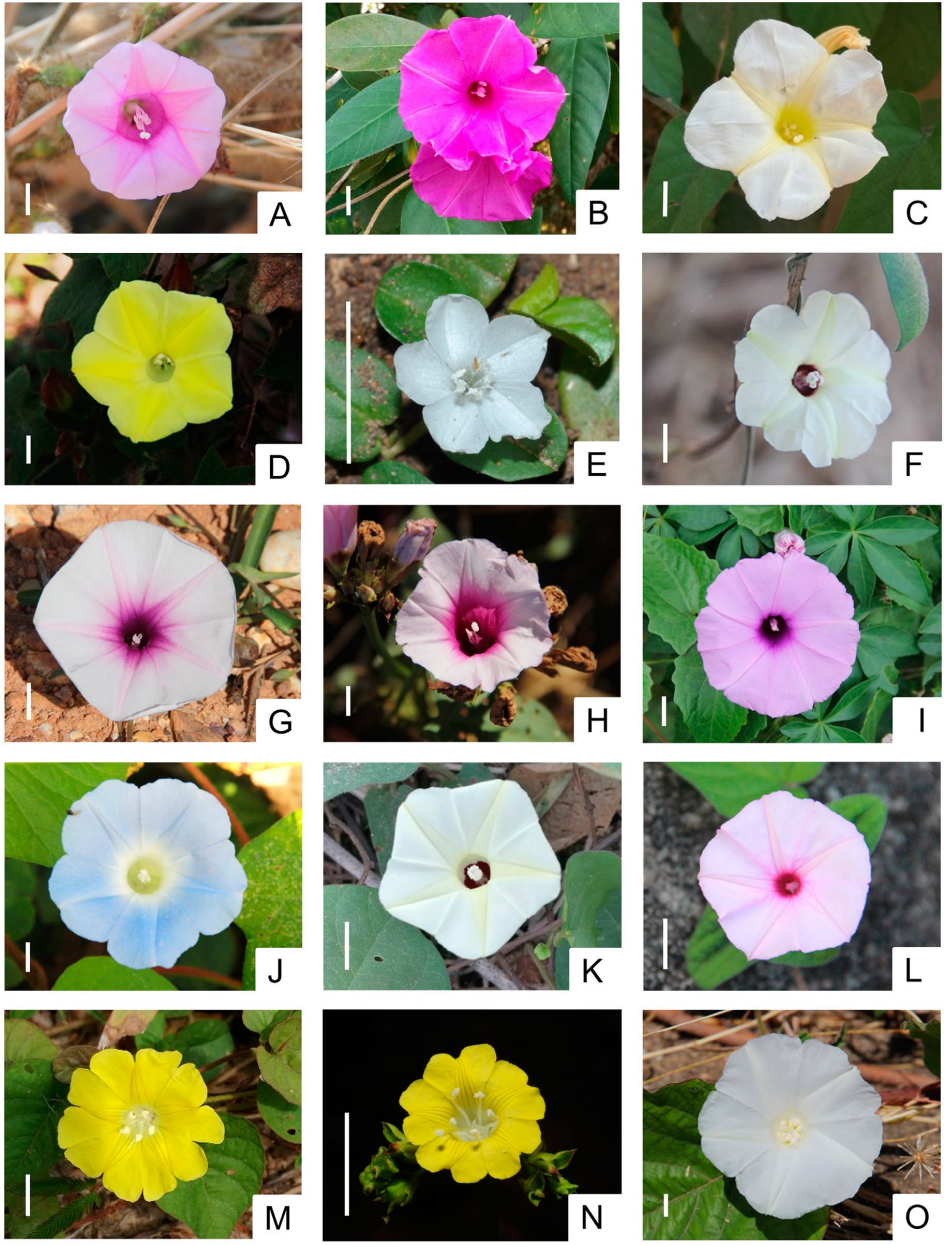
Photos of the 15 Convolvulaceae study species in Nong Khai province, Thailand. (**A**) *Argyreia capitiformis*, (**B**) *A. lanceolata*, (**C**) *Camonea pilosa*, (**D**) *C. vitifolia*, (**E**) *Evolvulus nummularius*, (**F**) *Hewittia malabarica*, (**G**) *Ipomoea aquatica*, (**H**) *I. batatas*, (**I**) *I. cairica*, (**J**) *I. nil*, (**K**) *I. obscura*, (**L**) *I. pileata*, (**M**) *Merremia gemella*, (**N**) *M. hederacea*, and (**O**) *Operculina turpethum.* Photo (**E**) was taken by Wittawat Kiewbang, (**N**) by Preecha Karaket, and all others by Piriya Hassa. [white scale bar = 1 cm]

We examined all Convolvulaceae species encountered in our study area, resulting in a total of 15 study species: *Argyreia capitiformis* (Poir.) Ooststr., *A. lanceolata* Choisy, *Camonea pilosa* (Houtt.) A.R. Simões & Staples, *C. vitifolia* (Burm.f.) A.R. Simões & Staples, *Evolvulus nummularius* (L.) L., *Hewittia malabarica* (L.) Suresh, *Ipomoea aquatica* Forssk., *I. batatas* (L.) Lam., *I. cairica* (L.) Sweet, *I. nil* (L.) Roth, *I. obscura* (L.) Ker Gawl., *I. pileata* Roxb., *Merremia gemella* (Burm.f.) Hallier f., *M. hederacea* (Burm.f.) Hallier f., and *Operculina turpethum* (L.) Silva Manso (Fig. 2). All plants were identified by Piriya Hassa and voucher specimens are stored in the public collection of the Plant Taxonomy Lab, Department of Plant Science, Faculty of Science, Mahidol University (see Supplementary Table 1 for voucher ID numbers). All of our study species are climbers, creepers, or herbaceous twiners with milky sap [23]. They have perfect flowers with pentamerous petals that are fused and radially symmetric, and corollas can be either funnelform, campanulate to funnelform, or campanulate [23]. Floral longevity ranges from approximately four hours in *H. malabarica* to approximately 13.5 h in *A. lanceolata* (Supplementary Table 1). Flowers have 2–4 stigmas with five stamens that are of variable length in some species [23, 43]. Each syncarpous pistil generally has two locules (rarely 3–5) in a superior ovary; each locule may have one or two ovules [23]. Our study species produce fruits that are either capsules or berries [23]. Dehiscent fruits are reported to carry 1–4 and occasionally up to six seeds [23]. Details of each study species, including flowering months and anthesis times, are included in Supplementary Table 1.

### Pollination experiments

We conducted pollination experiments to examine the dependence of Convolvulaceae species on pollinators, as well as to gain insight into their breeding systems. In each pollination experiment, five treatments were used: open pollination (flowers were not manipulated and were left exposed to all visitors), open emasculation (upon blooming, stamens were removed and flowers were then left exposed to all visitors), hand-cross pollination (flowers were pollinated by hand using xenogamous pollen and then covered with fine mesh bags to prevent visitation by pollinators), hand-self pollination (flowers were pollinated by hand using autogamous pollen and then covered with fine mesh bags), and the closed treatment (flowers were covered with fine mesh bags during the entire anthesis period). For each hand-cross pollination, we selected a pollen donor from the same population as the ovule donor but spaced 2–15 m apart. We collected fruits three weeks after pollination and counted seed set (number of seeds per fruit) to quantify the pollination success of each treatment. We conducted pollination experiments in 1–4 populations per plant study species (populations were at least 500 m apart; Supplementary Table 1), depending on the number of populations that we were able to find. There were two species (*A. lanceolata* and *I. pileata*) in which we were not able to conduct all five treatments. For *A. lanceolata* we only conducted the open and closed treatments because we found just seven flowering individuals, with only 1–2 flowers per plant. For *I. pileata* we did not conduct the open emasculation treatment because the narrow corolla tube made it impossible for us to cleanly remove the anthers (i.e., autogamous pollen always landed on the stigmas during our attempts to remove the anthers).

Four species (*A. capitiformis*, *A. lanceolata*, *I. cairica*, and *M. gemella*) had either no seed set or extremely low seed set. We therefore conducted additional pollination experiments during December 2022 – January 2023 for three of the species, but could not do so for *A. lanceolata* as we were not able to find any individuals in 2023. For *A. capitiformis*, *I. cairica*, and *M. gemella*, we conducted pollination experiments in three populations each, all at least 10 km apart. We used three treatments: open, hand-self pollination, and hand-cross-population pollination. The open and hand-self treatments were the same as during the initial round of pollination experiments. For each ovule donor used in the hand-cross-population pollinations, we used pollen donors from the other two populations. Thus, pollen from population A was used to pollinate flowers in populations B and C, pollen from population B was used to pollinate flowers in populations A and C, and pollen from population C was used to pollinate flowers in populations A and B. The hand-cross-population treatment allowed us to test whether the minimal seed set in the earlier experiments was due to low intra-population genetic diversity.

### Floral visitor observations

We observed floral visitors in 1–4 populations per plant study species (populations were at least 500 m apart; Supplementary Table 1). Animal visitors were recorded during anthesis using a video camera (Sony Handycam SR12); under dim or dark conditions, this model uses night-shot mode with infrared lighting. We recorded an average of 32 h of video footage per plant species (range: 6–52 h; Supplementary Table 1). When reviewing the video footage, animals were identified to the lowest taxonomic level possible using local field guides [44, 45] and with assistance from local entomologists (see Acknowledgements). No vertebrates were observed visiting the flowers. Some insects are easily identified to species (e.g., bees in the genus *Apis*, which have distinctive abdominal stripe patterns and colors), while others could only be identified to genus, tribe, family, or order (e.g., those that are species-rich, morphologically similar, and taxonomically unresolved, such as the halictid genus *Lasioglossum* and the stingless bee tribe Meliponini). Insect specimens were not collected to avoid disturbing subsequent animal visits and to avoid damaging flowers given the limited number of flowers for several study populations. Animals were categorized as visitor or potential pollinator based on their behavior. Animals that clearly contacted floral reproductive structures were scored as pollinators, while animals that visited that flower but did not contact the stigmas and anthers were scored as visitors. For plant species with broader corolla tubes (e.g., *H. malabarica*), cameras were positioned to ensure that contact with floral reproductive structures could be observed. For plant species with narrow corolla tubes and inserted stamens (e.g., *I. aquatica*), we assumed that all animals entering the corolla tube contacted floral reproductive structures. Both visitor and pollinator data were summarized, but only pollinator data were analyzed statistically.

### Statistical analysis

All analyses were conducted in R 4.2.2 [46]. For each study species, we used linear mixed modelling (LMM; package “lme4”) to analyze the pollination experiment data (using treatment as the fixed factor, plant ID as a random factor, and seed set as the response variable) and the pollinator observation data (using pollinator taxa as the fixed factor, plant ID as a random factor, and visitation rate as the response variable). Upon finding distinct patterns of pollinator dependence from our pollination experiment results, we also examined the relationship between pollinator dependence (fixed factor) and total pollinator visitation rate (response variable) using linear modelling (package “stats”). With each set of analyses, models were compared with nested likelihood ratio tests and Tukey’s post-hoc test (package “emmeans”) was performed when the fixed factor was significant.

## Results

### Pollination experiments

**Fig. 3:**
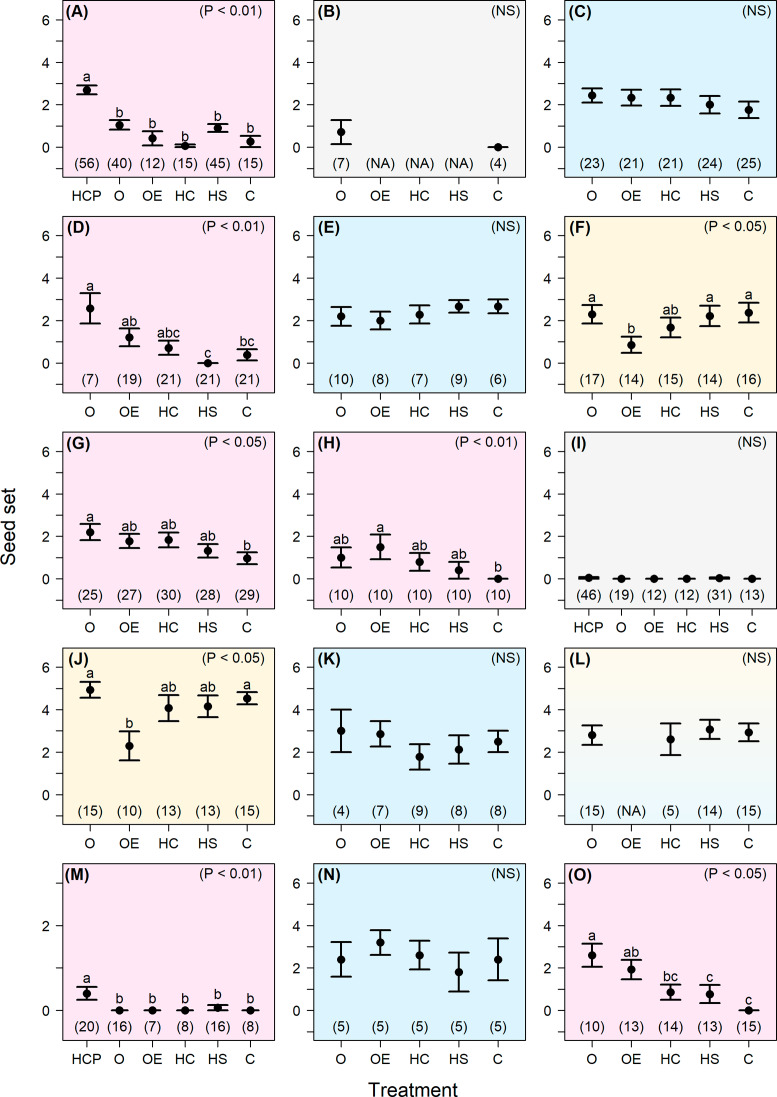
Seed set in the 15 Convolvulaceae study species in Nong Khai province, Thailand. Average seed set (mean number of seeds per fruit ± SE) results from pollination experiments comparing different treatments (HCP: hand-cross-population pollination; O: open; OE: open emasculation; HC: hand cross-pollination; HS: hand self-pollination; C: closed) in 15 Convolvulaceae species in Nong Khai, Thailand: (**A**) *Argyreia capitiformis*, (**B**) *A. lanceolata*, (**C**) *Camonea pilosa*, (**D**) *C. vitifolia*, (**E**) *Evolvulus nummularius*, (**F**) *Hewittia malabarica*, (**G**) *Ipomoea aquatica*, (**H**) *I. batatas*, (**I**) *I. cairica*, (**J**) *I. nil*, (**K**) *I. obscura*, (**L**) *I. pileata*, (**M**) *Merremia gemella*, (**N**) *M. hederacea*, and (**O**) *Operculina turpethum*. Colors represent different degrees of pollinator dependence: blue = low dependence and receive high amounts of cross pollen (C, E, K, N); yellow = low dependence and receive low amounts of cross pollen (F, J); pink = high dependence (A, D, G, H, M, O); grey = inconclusive (B, I). [Note: *Ipomoea pileata* is shown as blue and yellow because it exhibits low dependence but amounts of cross pollen are unknown.] Treatments with different letters have significantly different seed set (Tukey’s post-hoc; p-values in the upper right corner of each graph). Sample sizes (n) listed above each treatment refer to the number of plants used in the experiment

Our pollination experiment results revealed that most of our study species are self-compatible to varying degrees (Fig. 3). In 11 species, the seed set of hand-self pollinated and hand-cross pollinated treatments were not significantly different (i.e., species are self-compatible). *Camonea vitifolia* did appear to set fewer seeds in the hand-self pollinated treatment compared to the hand-cross pollinated treatment, however, the difference was not statistically significant. For the other four study species, results were less conclusive. *Argyreia capitiformis* appears to exhibit inter-population variation in either self-compatibility or in genetic load. In one *A. capitiformis* population the hand-self treatment set as many seeds as the hand-cross-population treatment, while in two other populations the hand-self treatment set significantly fewer seeds than the hand-cross-population treatment (Supplementary Fig. 1). *Merremia gemella* did not set seed in any treatments in three populations, while there was some seed set in the hand-cross-population treatment in a fourth population (Supplementary Fig. 1). We could not assess the compatibility systems of *A. lanceolata* and *I. cairica* as they set few to no seeds in each treatment.

Most of our study species also appear to not be mate- or pollen-limited, with the open pollination treatment producing at least as many seeds as the hand-cross pollinated treatment (Fig. 3). However, *A. capitiformis* and *M. gemella* appear to be mate-limited in most or all of their populations; intra-population crosses rarely (for *A. capitiformis*) or never (for *M. gemella*) set seed, and hand-cross-population treatments set significantly more seed than open treatments for some of their populations (Supplementary Fig. 1).

In terms of pollinator dependency, three main patterns were observed from our pollination experiments. In the first pattern, high seed set occurred in all treatments, and these species were categorized as having low dependence on pollinators with high outcrossing rates (blue panels in Fig. 3). In the second pattern, there was lower seed set in the open emasculation treatment compared to the other treatments, and these species were categorized as having low dependence on pollinators with low outcrossing rates (yellow panels in Fig. 3). In the third pattern, there was lower seed set in the closed treatment compared to the open or open emasculation treatments, and these species were categorized as having high dependence on pollinators (pink panels in Fig. 3). There were also two species that had low seed set across all treatments, and these species were categorized as a fourth pattern: degree of pollinator dependence inconclusive (grey panels in Fig. 3).

In the first pattern (low dependence on pollinators with high outcrossing rates; blue panels in Fig. 3), there were no significant differences among treatments (Supplementary Table 2) because seed set was relatively high in all treatments tested (generally around 2–3 seeds per fruit). Four species exhibited this pattern: *C. pilosa* (Fig. 3C), *E. nummularius* (Fig. 3E), *I. obscura* (Fig. 3K), and *M. hederacea* (Fig. 3N). In the second pattern (low dependence on pollinators with low outcrossing rates; yellow panels in Fig. 3), there were significant differences among treatments (Supplementary Table 2), with the open emasculation treatment setting fewer seeds than the other treatments, as seen in *H. malabarica* (Fig. 3F) and *I. nil* (Fig. 3J). In the third pattern (high dependence on pollinators; pink panels in Fig. 3), there were significant differences among treatments (Supplementary Table 2), with the closed treatment setting fewer seeds than the open, open emasculation, or hand-cross-population treatments. Six species exhibited this third pattern: *A. capitiformis* (Fig. 3A), *C. vitifolia* (Fig. 3D), *I. aquatica* (Fig. 3G), *I. batatas* (Fig. 3H), *M. gemella* (Fig. 3M), and *O. turpethum* (Fig. 3O). In the fourth pattern (degree of pollinator dependence inconclusive; grey panels in Fig. 3), there were no significant differences among treatments (Supplementary Table 2) because seed set was low in all treatments tested (0–1 seeds per fruit). Two species exhibited this pattern: *A. lanceolata* (Fig. 3B) and *I. cairica* (Fig. 3I). One species (*I. pileata*, Fig. 3L) had high seed set in all four treatments examined, but we could not determine whether it exhibited pattern one or pattern two since we were not able to collect data for the open emasculation treatment.

### Floral visitor observations

In total, we observed 46 animal taxa from seven orders visiting the flowers of our study species, but only 37 taxa representing seven orders were classified as potential pollinators (Supplementary Table 3) based on their observed contact with floral anthers and stigmas. Most taxa classified as pollinators belonged to the orders Hymenoptera (bees, wasps, and ants; 13 taxa) and Lepidoptera (butterflies and moths; 16 taxa). The remaining taxa (Blattodea, Coleoptera, Diptera, Hemiptera, and Orthoptera) were infrequent and generally classified only to the level of order. When pooling the visits of all pollinators of each plant study species, total pollinator visitation rates ranged from 40.98 ± 15.84 visits per hour in *M. hederacea* to 0.67 ± 0.20 visits per hour in *I. pileata* (Supplementary Table 4).

**Fig. 4:**
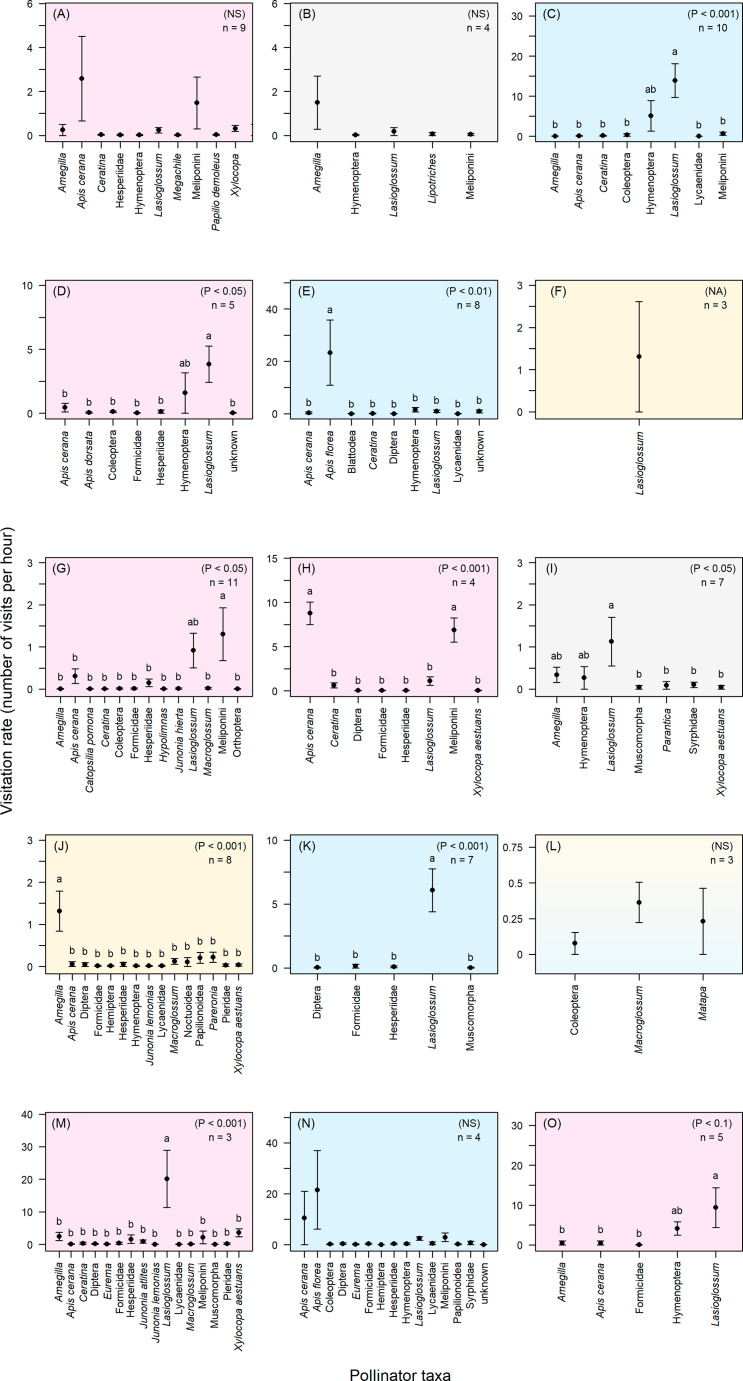
Pollinator visitation to the 15 Convolvulaceae study species in Nong Khai province, Thailand. Mean (± SE) visitation rates (number of visits per hour) of pollinator taxa observed at each of the 15 Convolvulaceae study species in Nong Khai, Thailand. (**A**) *Argyreia capitiformis*, (**B**) *A. lanceolata*, (**C**) *Camonea pilosa*, (**D**) *C. vitifolia*, (**E**) *Evolvulus nummularius*, (**F**) *Hewittia malabarica*, (**G**) *Ipomoea aquatica*, (**H**) *I. batatas*, (**I**) *I. cairica*, (**J**) *I. nil*, (**K**) *I. obscura*, (**L**) *I. pileata*, (**M**) *Merremia gemella*, (**N**) *M. hederacea*, and (**O**) *Operculina turpethum*. Colors represent different degrees of pollinator dependence: blue = low dependence and receive high amounts of cross pollen (C, E, K, N); yellow = low dependence and receive low amounts of cross pollen (F, J); pink = high dependence (A, D, G, H, M, O); grey = inconclusive (B, I). [Note: *Ipomoea pileata* is shown as blue and yellow because it exhibits low dependence but amounts of cross pollen are unknown.] All individuals observed contacting floral reproductive structures were classified as potential pollinators and included in the graphs. Pollinator taxa with different letters have significantly different visitation rates; for the values in the upper right-hand corner of each graph, the p-values were obtained from Tukey’s post-hoc tests and the sample sizes (n) refer to the number of plants observed

When comparing visitation rates among pollinator taxa, our results from linear mixed modelling showed that 10 species exhibited significant differences in pollinator visitation rates, namely, *C. pilosa*, *C. vitifolia*, *E. nummularius*, *I. aquatica, I. batatas*, *I. cairica*, *I. nil, I. obscura*, *M. gemella*, and *O. turpethum* (Fig. 4; Table 1). In contrast, four plant species did not exhibit significant differences in pollinator visitation rates (*A. capitiformis*, *A. lanceolata*, *I. pileata*, and *M. hederacea*). One species (*H. malabarica*) could not be tested as it was visited by a single pollinator taxon (Table 1; Fig. 4). All 15 species received the majority of floral visitations from 1 to 2 pollinator taxa (Table 1; Fig. 4). Seven study species were visited primarily by *Lasioglossum* species (Halictidae, Curtis, 1833), two depended on *Amegilla* species (Apidae, Friese, 1897), and two depended on *Apis florea* (Apidae, Fabricius, 1787). Two other study species were visited by both *Apis cerana* (Apidae, Fabricius, 1793) and unidentified species in the tribe Meliponini (Apidae, Lepeletier, 1836). One study species received visits from *Lasioglossum* species and members of the Meliponini (Table 1). The only species that was not visited at all by bees appeared to depend on crepuscular-nocturnal Lepidoptera and was visited by hawkmoth species in the genus *Macroglossum* (Scopoli, 1777) and a large butterfly in the genus *Matapa* (Moore, 1881; Table 1).

**Table 1:** Statistical results of pollinator observations for the 15 Convolvulaceae study species

Plant species	LMM			Tukey’s	Main Pollinator(s)
	Chi-square	df	P	P	
*Argyreia capitiformis*	16.34	10	0.105	NS	*Apis cerana* Meliponini
*Argyreia lanceolata*	7.70	5	0.193	NS	*Amegilla*
*Camonea pilosa*	42.03	8	**< 0.001**	**< 0.001**	*Lasioglossum*
*Camonea vitifolia*	22.06	8	**0.003**	**< 0.05**	*Lasioglossum*
*Evolvulus nummularius*	32.26	10	**0.001**	**< 0.01**	*Apis florea*
*Hewittia malabarica*	N/A	N/A	N/A	N/A	*Lasioglossum*
*Ipomoea aquatica*	64.14	11	**< 0.001**	**< 0.05**	*Lasioglossum* Meliponini
*Ipomoea batatas*	70.00	7	**< 0.001**	**< 0.001**	*Apis cerana* Meliponini
*Ipomoea cairica*	15.92	6	**0.014**	**< 0.05**	*Lasioglossum*
*Ipomoea nil*	93.71	19	**< 0.001**	**< 0.001**	*Amegilla*
*Ipomoea obscura*	62.60	8	**< 0.001**	**< 0.001**	*Lasioglossum*
*Ipomoea pileata*	2.07	2	0.355	NS	*Macroglossum* *Matapa*
*Merremia gemella*	104.00	28	**< 0.001**	**< 0.001**	*Lasioglossum*
*Merremia hederacea*	39.75	24	0.072	NS	*Apis florea*
*Operculina turpethum*	15.33	5	**0.023**	< 0.1	*Lasioglossum*

Results (chi-square values, degrees of freedom, and p-values) of linear mixed models (LMM) and Tukey’s post-hoc tests for each of 15 Convolvulaceae species examining significant differences among pollinator visitation rates (significant p-values are in bold). The pollinator taxa that visited the most often are also listed. LMM could not be performed for *H. malabarica* because only a single pollinator taxon was observed

**Fig. 5:**
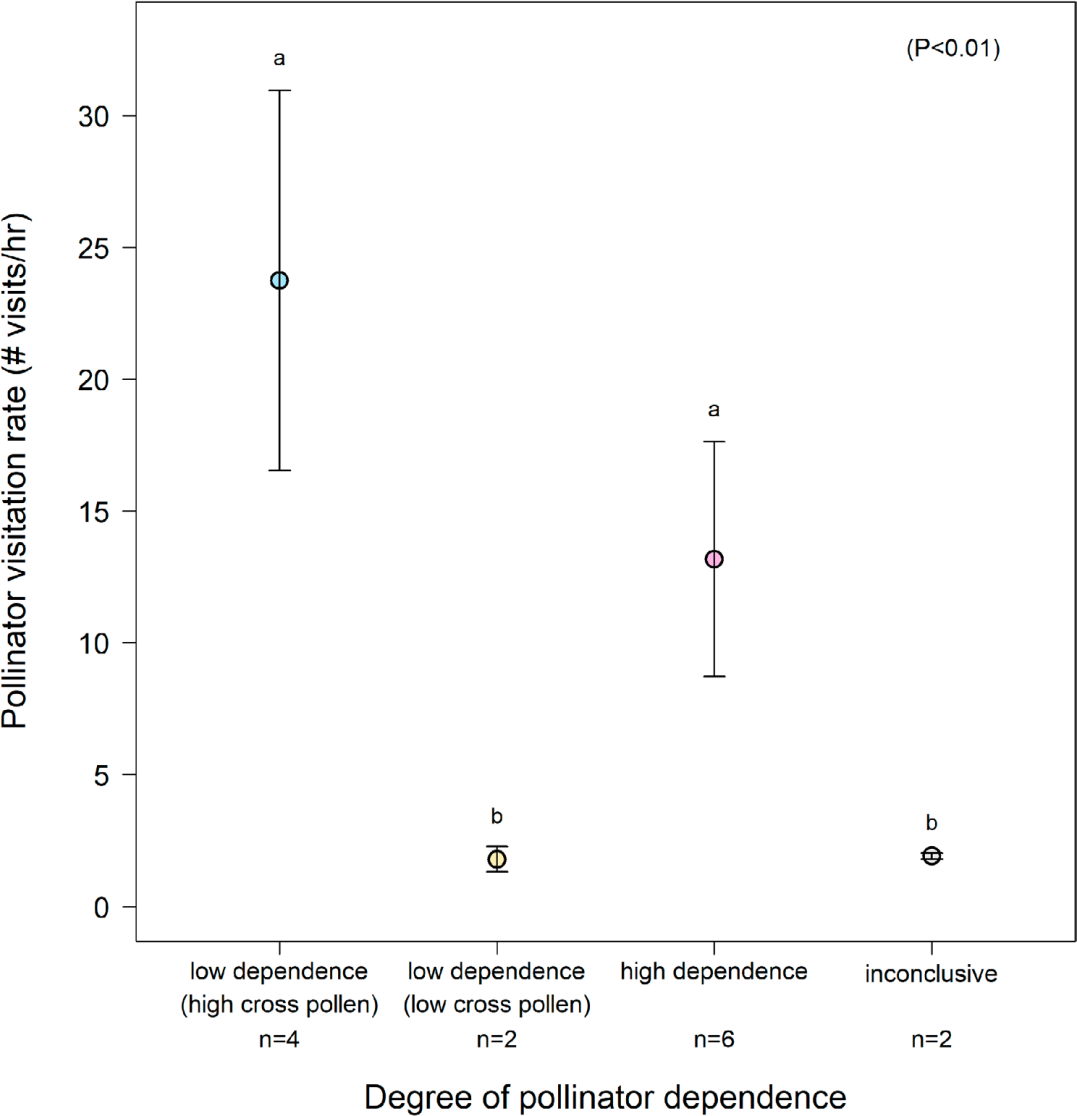
Association between degree of pollinator dependence and pollinator visitation rates. Mean (± SE) visitation rates (number of visits per hour) of Convolvulaceae study species in Nong Khai, Thailand categorized by degree of pollinator dependence (and how much xenogamous pollen is received). Categories with different letters have significantly different pollinator visitation rates (Tukey’s post-hoc, P < 0.01). Sample sizes (n) indicate the number of Convolvulaceae species classified in each category. [Note: *Ipomoea pileata* could not be definitively categorized and was therefore excluded.]

We also observed significant differences in overall pollinator visitation rates for each of the four patterns of pollinator dependence (${\text{{\rm X}}}_{3}^{2}$ = 17.49, P < 0.001; Fig. 5). Plant species exhibiting pattern one (low dependence on pollinators with high outcrossing rates) or pattern three (high dependence on pollinators) had significantly higher visitation rates from prospective pollinators than plant species exhibiting pattern two (low dependence on pollinators with low outcrossing rates) or pattern four (degree of pollinator dependence inconclusive; Fig. 5).

## Discussion

### Compatibility systems and pollinator dependency in some species in the Convolvulaceae of Thailand

Most of our study species showed varying degrees of self-compatibility. Martin [26] also reported that self-compatibility is more common than self-incompatibility in this family (38 vs. 13 species, respectively). McDonald et al. [36] estimated 16 independent origins of autogamy in *Ipomoea* L., with only four retrograde transitions to xenogamy. Delgado-Dávila & Martén-Rodríguez [18] examined self-compatible *Ipomoea hederacea* Jacq. and found that self-pollination was beneficial due to its reproductive assurance and outweighed the costs of cross-pollination as inbreeding depression was limited in this species. Self-compatibility appears to be very common in the Convolvulaceae, paralleling other large, angiosperm lineages dominated by animal-pollinated species including the Orchidaceae [47] (but see [19]), the Commelinaceae [48], *Heliconia* L. (Heliconiaceae) [49], and *Rosa* L. (Rosaceae) [50].

The prevalence of self-compatibility in the Convolvulaceae may be due in part to selection for reproductive assurance [10, 18] and we did find evidence for this in seven of our study species. This reproductive assurance should be particularly beneficial for the two study species that received few pollinator visits and had low outcrossing rates. However, the six self-compatible study species exhibiting high dependence on pollinators appear to have a far weaker or no reproductive assurance as their flowers failed to fructify in the absence of pollinators. If self-compatibility is ancestral in these taxa they may not be under selection to evolve reproductive assurance via autonomous self-pollination as their pollinators visit frequently over their brief, floral life-spans (~ 13 visits per hour, on average). We acknowledge that further research is necessary to examine spatial and temporal variation in pollinator visitation [51, 52].

We also note that one of our study species appears to exhibit inter-population variation in either self-compatibility or genetic load. The three *A. capitiformis* populations examined in 2017–2019 set very few seeds, leading us to hypothesize that this species is self-incompatible and mate-limited with low genetic diversity at the population level. However, our results in 2023 showed that hand-self-pollinated flowers could set seed in two populations, although significantly fewer compared to hand-crosses between populations. Moreover, all pollination treatments of *A. capitiformis* in the third population set comparable numbers of seeds (Supplementary Fig. 1). These results suggest that either levels of self-incompatibility vary among populations, or genetic load and levels of inbreeding depression vary across the same populations. All hand-cross-population treatments for *A. capitiformis* had relatively high seed set (2–4 seeds per fruit), indicating a higher inter-population than intra-population genetic diversity. Intraspecific variation in self-compatibility [17, 53] and inbreeding depression [54] have been reported for other plant species in other angiosperm lineages.

Three other study species (*A. lanceolata*, *I. cairica*, and *M. gemella*) had low seed set across all treatments, which may be due to a number of factors. Insufficient pollinator visitation is an unlikely explanation as all three species received at least three visits per hour, but low pollen quantity and/or quality may have contributed to their poor seed set [55]. Indeed, we observed only seven flowering individuals of *A. lanceolata* in our study area, so while our plants were not pollinator-limited, they may have been mate-limited [56]. As they are all short-lived flowers, there is also the possibility that some hand pollinations across individuals and populations were made when stigmas were not receptive, resulting in low fertilization success. Another possible explanation may be that our populations are largely self-incompatible and have low genetic diversity (e.g., clonal populations stemming from vegetative growth) [57]. This explanation is supported by previous references to self-incompatibility in *I. cairica* [39] and reports that animal-pollinated flowers of *I. cairica* in Brazil do set fruit [35]. In contrast, our study populations may be self-compatible but have high inbreeding depression, resulting in poor reproductive success for genetically depauperate populations [58, 59]. A final explanation could be the presence of pollen, stylar, and/or ovarian sterility, as reported previously for *Ipomoea batatas* (L.) Lam. [60], *Ipomoea pandurata* (L.) G.F.W. Meyer [61], and *Ipomoea wolcottiana* Rose [62]. As inter- and intra-population variability in reproduction has been observed repeatedly in the Convolvulaceae [61, 62], further research examining multiple populations is necessary to determine the causes of low seed set in *A. lanceolata*, *I. cairica*, and *M. gemella* in Nong Khai, Thailand.

Our study species also exhibited different degrees of pollinator dependency. We hypothesize that these differences in pollinator dependence are due to the relative positions of stigmas and anthers (i.e., herkogamy). Indeed, the importance of anther-stigma distance on rates of autonomous self-pollination is well known [63–65], including in the Convolvulaceae [28, 36–38, 66]. In our study, the six self-compatible species that experienced reduced seed set in the closed treatment may have high levels of herkogamy. Substantial spatial separation between stigmas and anthers could preclude mechanical self-pollination in the absence of floral visitors [65]. In contrast, the seven self-compatible species with high seed set in the closed treatment may have stamens and pistils that are more similar in length, enabling autogamous pollen to reach the stigmas in the absence of floral visitors [65]. Informal observation of our study species showed that, overall, the six self-compatible species showing a higher dependency on pollinators appear to exhibit greater herkogamy than the seven self-compatible species showing a lower dependency on pollinators. However, systematic observation examining numerous individuals is still needed, since stigma-anther separation is known to vary at intraspecific and temporal levels [18, 37, 65].

### Pollinators of Thai Convolvulaceae

Bees were the most frequent of floral visitors to our plant study species. Five bee taxa belonging to two families (Apidae and Halictidae) were dominant visitors to 14 out of 15 of our study species. The pollinator taxa observed at each species corresponds to their flowering times. All of our study species visited primarily bees began blooming during the early morning (generally 06h00–08h00) and flowers wilted by late afternoon or early evening (Supplementary Table 1). In contrast, flowers of *I. pileata* bloomed from 15h30 to 02h00 (Supplementary Table 1) and were visited primarily by crepuscular insects in the order Lepidoptera. Our results are also consistent with previous studies that have reported bees as the main pollinators of convolvulaceous species [29, 31, 33–35, 39–41, 61], although some species are reported to depend on butterflies [41], hawkmoths [29, 31, 32], bats [31], or hummingbirds [29, 31].

As flowers of our study species receive the majority of visits from one to two taxa in one or two families in the same insect order, it appears we are observing specialized pollination systems [67]. Limited pollinator diversity may confer an increased risk of low or variable pollination success [68]. A meta-analysis by Knight et al. [15] found that pollen limitation was greater for species pollinated by fewer animal taxa. Moreover, plants receiving visits from fewer pollinator guilds tend to experience greater frequencies of autonomous selfing, supporting the reproductive assurance hypothesis [69]. While our plant study species appear to have specialized pollination systems, they are visited by generalist bees known to forage on diverse plant taxa [70, 71]. Prior research examining plant-pollinator interactions shows that specialist species tend to interact with generalist species, which helps buffer against species extinctions [72]. It is important to note that our classification of pollinators was based solely on our observations of which insects contacted stigmas and anthers. Additional field and lab studies are needed to confirm whether these insects carry pollen of these host flowers in Thailand and deposit them on stigmas when they are biochemically receptive.

Two of our study species provide insight into how pollinators can vary globally. *Ipomoea cairica* and *I. nil* have been introduced widely around the world and their pollinators been reported in neotropical countries. In southeastern Brazil, flowers of *I. cairica* were visited by 16 bee taxa and nine of them were classified as efficient pollinators [35]. In Argentina, flowers of the same species were visited by seven bee taxa including two *Bombus* species (Apidae) [29]. Our study in northeastern Thailand found that, while *I. cairica* was visited primarily by bees and wasps, *Lasioglossum* species were the most frequent visitors. *Ipomoea nil* was visited by 11 bee taxa in southeastern Brazil, and six were classified as efficient pollinators [35]. In northeastern Brazil, flowers of *I. nil* were visited by 10 bee taxa and one butterfly species, but 80% of visits were by *Lithurgus huberi* in the family Megachilidae [33]. Our study found that while the potential pollinators of *I. nil* are diverse, *Amegilla* bees were by far the most frequent visitors. Consequently, while some species within the Convolvulaceae continue to rely primarily on bee pollinators no matter where they are introduced, their reproductive success may not be co-adapted to a specific bee genus or lineage.

## Conclusions

The results of this study show that some species within the Convolvulaceae are highly dependent on pollinators to set seed, while others are capable of autonomous selfing (spontaneous autogamy) in the absence of insect vectors of pollen. As most of our study species were found to be self-compatible, we hypothesize that this variation in pollinator dependence is due to different degrees of herkogamy and species exhibiting broader herkogamy are more dependent on pollinators. Those species exhibiting high dependence on pollinators (e.g., self-incompatible species and herkogamous species) may be more vulnerable to environmental changes that impact their pollinators. Species capable of autonomous selfing, while not dependent on pollinators to set seed, continue to benefit from cross-pollination, as all of our study species received visitations from pollinators. However, based on subsequent fruit and seed set, some species appear to have received lower amounts of xenogamous pollen due to fewer pollinator visits, indicating lower gene flow between and within populations. All but one of our study species were pollinated primarily by bees representing the families Apidae and Halictidae. The lone exception attracted crepuscular visitors in the order Lepidoptera. The cumulative findings of this study indicate that pollinator dependence is influenced by breeding system, and suggest that pollinator visitation is consistently higher for species exhibiting high pollinator dependence but varies across species exhibiting low pollinator dependence. The findings of this study are also important for assessing future conservation risks of paleotropical members of the Convolvulaceae and their response to environmental changes that affect the demography and diversity of their pollinators.

### Electronic supplementary material

Below is the link to the electronic supplementary material.

Supplementary Material 1

## Data Availability

All data are available at https://data.mendeley.com/datasets/wpv6jx3yc4 (doi: 10.17632/wpv6jx3yc4.1).
