# Supplementary material for: Compatibility systems and pollinator dependency in morning glory species (Convolvulaceae)
Source: BMC Plant Biol. 2023 Sep 15;23:432. doi: 10.1186/s12870-023-04437-y (PMC10503090; doi:10.1186/s12870-023-04437-y)
Supplement: Supplementary file 1 — Supplementary Material 1 [file 12870_2023_4437_MOESM1_ESM.docx]

**SUPPLEMENTARY INFORMATION**

**Compatibility systems and pollinator dependency in morning glory species (Convolvulaceae)**

Piriya Hassa, Paweena Traiperm, and Alyssa B. Stewart


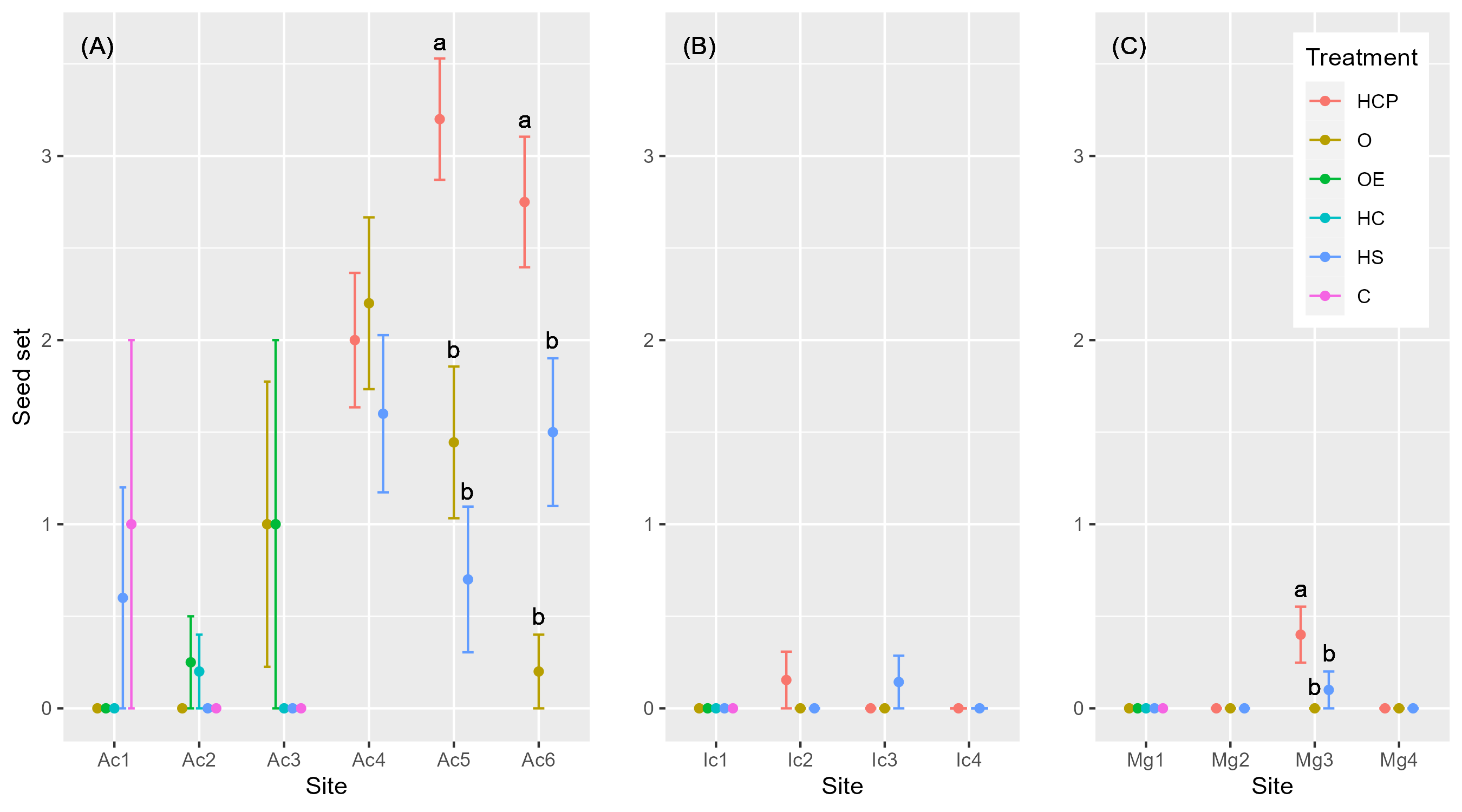


**Supplementary Figure 1**. Results of average seed set (mean number of seeds per fruit ± SE) from pollination experiments on three species (A, *Argyreia capitiformis*; B, *Ipomoea cairica*; C. *Merrenia gemella*) in Nong Khai Thailand conducted at different sites comparing different treatments. HCP: hand-cross-population pollination, red; O: open, yellow; OE: open emasculation, green; HC: hand cross-pollination, teal; HS: hand self-pollination, blue; C: closed, pink). These treatments were conducted to confirm whether the minimal seed set in earlier experiments was due to low intra-population genetic diversity of self-incompatible species. Within each site, treatments with different letters have significantly different seed set (Tukey’s post-hoc, P < 0.05).

Supplementary Table 1. Comparative information on populations representing 15 species in the Convolvulaceae in Nong Khai, Thailand including the number of video hours recorded for pollination observations and when pollination experiments were conducted (R1 = December 2017 – March 2019, R2 = December 2022 – January 2023). Voucher ID numbers are included in parentheses under population coordinates where applicable.

| **Species** | **Anthesis Time** | **Flowering phenology** | **Population coordinates**  **(voucher ID)** | **Population area (m^2^)** | **Habitat** | **pollinator observation** | **pollination experiment** |
| --- | --- | --- | --- | --- | --- | --- | --- |
| *Argyreia capitiformis* | 06.00-17.00 | Dec - Apr | 18°0'28.8"N  102°19'53.4"E  (P. Hassa 13) | 900 | rubber plantation | 4:16 h | R1 |
|  |  |  | 18°1'15.2"N  102°20'36.3"E | 100 | roadside | 4:04 h | R1 |
|  |  |  | 17°47'03.2"N 102°36'01.5"E | 10,000 | roadside | - | R2 |
|  |  |  | 17°55'34.3"N 102°36'23.5"E | 900 | abandoned lot | - | R2 |
|  |  |  | 18°00'34.5"N 102°19'50.2"E | 10,000 | thickets | - | R2 |
| *Argyreia lanceolata* | 5.30-19.00 | Oct - Dec | 18°0'24.4"N  102°17'14.3E  (P. Hassa 6) | 2,500 | forest | 27:31 h | R1 |
| *Camonea pilosa* | 7.00-15.00 | Feb - Apr | 17°40'57.4"N  102°44'12.4"E | 10,000 | rice paddies | 3:49 h | R1 |
|  |  |  | 17°40'58.4"N  102°44'10.5"E | 400 | rice paddies | 8:26 h | R1 |
|  |  |  | 17°57'58.3"N  102°30'57.2"E  (P. Hassa 25) | 10,000 | thickets | 7:41 h | R1 |
|  |  |  | 17°58'0.5"N  102°30'42.0"E | 900 | tobacco field | 11:27 h | R1 |
| *Camonea vitifolia* | 7.30-16.30 | Dec - Mar | 17°57'44.1"N  102°31'48.6"E  (P. Hassa 19) | 900 | abandoned lot | 12:11 h | R1 |
|  |  |  | 17°58'0.4"N  102°30'41.3"E  (P. Hassa 21) | 1600 | pineapple field | 16:49 h | R1 |
| *Evolvulus nummularius* | 7.00-13.00 | Jan - Dec | 17°46'47.2"N  102°48'3.1"E  (P. Hassa 29) | 400 | rice paddies | 27:41 h | R1 |
| *Hewittia malabarica* | 10.00-14.00 | Dec - Jan | 18°0'24.0"N  102°19'14.6"E  (P. Hassa 20) | 225 | roadside | 3:39 h | R1 |
|  |  |  | 18°0'28.8"N  102°19'53.4"E  (P. Hassa 26) | 100 | rubber plantation | 2:32 h | R1 |
| *Ipomoea aquatica* | 5.45-19.00 | Oct - Jan | 17°46'26.7"N  102°46'15.2"E  (P. Hassa 30) | 25 | garden | 23:21 h | R1 |
|  |  |  | 18°0'22.4"N  103°18'18.3"E  (P. Hassa 32) | 400 | rice paddies | 18:57 h | R1 |
| *Ipomoea batatas* | 6.30-17.00 | Nov - Feb | 18°5'41.0"N  102°8'41.5"E  (P. Hassa 24) | 225 | rice paddies | 28:46 h | R1 |
| *Ipomoea cairica* | 5.30-16.30 | Jan - Dec | 17°41'4.0"N  102°45'9.8"E  (P. Hassa 8) | 900 | rice paddies | 21:09 h | R1 |
|  |  |  | 17°41'03.8"N 102°45'12.2"E | 900 | abandoned lot | - | R2 |
|  |  |  | 17°49'43.9"N 102°35'59.7"E | 10,000 | roadside | - | R2 |
|  |  |  | 17°49'38.9"N 102°42'17.8"E | 900 | abandoned lot | - | R2 |
| *Ipomoea nil* | 4.00-14.00 | Jul - Sep | 17°44'32.6"N  102°41'1.1"E  (P. Hassa 16) | 900 | abandoned lot | 5:30 h | R1 |
|  |  |  | 17°49'3.9"N  102°36'13.5"E  (P. Hassa 4) | 1,600 | rice paddies | 46:41 h | R1 |
| *Ipomoea obscura* | 8.00-14.30 | Jan - Dec | 17°49'33.0"N  102°46'18.5"E  (P. Hassa 37) | 100 | orchard | 28:00 h | - |
|  |  |  | 17°58'0.4"N  102°30'41.3"E | 225 | pineapple field | - | R1 |
|  |  |  | 17°58'0.5"N  102°30'42.0"E | 100 | tobacco field | - | R1 |
| *Ipomoea pileata* | 15.30-02.00 | Jan - Apr | 18°0'21.5"N  102°20'10.8"E  (P. Hassa 26) | 900 | thickets | 25:39 h | R1 |
|  |  |  | 18°0'30.8"N  102°20'0.1"E | 400 | rubber plantation | - | R1 |
| *Merremia gemella* | 7.00-17.00 | Jan - Apr | 17°49'33.0"N  102°46'18.5"E | 10,000 | pond | 28:39 h | R1 |
|  |  |  | 17°47'11.5"N 102°36'02.5"E | 100 | abandoned lot | - | R2 |
|  |  |  | 17°50'17.5"N 102°42'23.0"E | 900 | abandoned lot | - | R2 |
|  |  |  | 17°53'15.5"N 102°30'21.7"E  (P. Hassa 50) | 10,000 | pond | - | R2 |
| *Merremia hederacea* | 8.00-13.00 | Nov - Jan | 18°0'1.4"N  103°18'13.5"E | 400 | space | 13:20 h | - |
|  |  |  | 18°0'22.4"N  103°18'18.3"E | 400 | rice paddies | 24:08 h | - |
|  |  |  | 18°5'41.8"N  102°8'51.5"E  (P. Hassa 51) | 400 | space | - | R1 |
| *Operculina turpethum* | 7.00-16.00 | Mar - May | 17°57'45.3"N  102°31'25.5"E | 900 | abandoned lot | 5:26 h | R1 |
|  |  |  | 17°58'0.5"N  102°30'42.0"E  (P. Hassa 33) | 10,000 | tobacco field | 9:00 h | R1 |

Supplementary Table 2. Seed set results of linear mixed models (LMM; chi-square values, degrees of freedom, and p-values) comparing significant differences between pollination treatments conducted for each species in Nong Khai, Thailand.

| **Species** | **Seed Set** | | |
| --- | --- | --- | --- |
|  | **Chi-sq** | **df** | **P** |
| *Argyreia capitiformis* | 16.303 | 5 | <0.001 |
| *Argyreia lanceolata* | 1.016 | 1 | 0.314 |
| *Camonea pilosa* | 7.153 | 4 | 0.128 |
| *Camonea vitifolia* | 23.468 | 4 | <0.001 |
| *Evolvulus nummularius* | 2.396 | 4 | 0.663 |
| *Hewittia malabarica* | 14.222 | 4 | 0.007 |
| *Ipomoea aquatica* | 12.930 | 4 | 0.012 |
| *Ipomoea batatas* | 15.826 | 4 | 0.003 |
| *Ipomoea cairica* | 0.268 | 5 | 0.930 |
| *Ipomoea nil* | 14.089 | 4 | 0.007 |
| *Ipomoea obscura* | 5.542 | 4 | 0.236 |
| *Ipomoea pileata* | 0.111 | 3 | 0.991 |
| *Merremia gemella* | 2.155 | 5 | <0.001 |
| *Merremia hederacea* | 2.457 | 4 | 0.652 |
| *Operculina turpethum* | 31.498 | 4 | <0.001 |

**Supplementary Table 3.** Arthropod taxa observed contacting floral stigmas and anthers of each of the 15 study species as indicated by tick marks: (A) *A. capitiformis*, (B) *A. lanceolata*, (C) *C. pilosa*, (D) *C. vitifolia*, (E) *E. nummularius*, (F) *H. malabarica*, (G) *I. aquatica*, (H) *I. batatas*, (I) *I. cairica*, (J) *I. nil*, (K) *I. obscura*, (L) *I. pileata*, (M) *M. gemella*, (N) *M. hederacea*, and (O) *O. turpethum*.

| **Arthropod Visitors** | | **Morning glory species** | | | | | | | | | | | | | | |
| --- | --- | --- | --- | --- | --- | --- | --- | --- | --- | --- | --- | --- | --- | --- | --- | --- |
| **Order** | **Taxa** | **A** | **B** | **C** | **D** | **E** | **F** | **G** | **H** | **I** | **J** | **K** | **L** | **M** | **N** | **O** |
| Blattodea | Blattodea (unidentified) |  |  |  |  | / |  |  |  |  |  |  |  |  |  |  |
| Coleoptera | Coleoptera (unidentified) |  |  | / | / |  |  | / |  |  |  |  | / |  | / |  |
| Diptera | Diptera (unidentified) |  |  |  |  | / |  |  | / |  | / | / |  | / | / |  |
|  | *Muscomorpha* |  |  |  |  |  |  |  |  | / |  | / |  | / |  |  |
|  | Syrphidae |  |  |  |  |  |  |  |  | / |  |  |  |  | / |  |
| Hemiptera | Hemiptera (unidentified) |  |  |  |  |  |  |  |  |  | / |  |  |  | / |  |
| Hymenoptera | *Amegilla* | / | / | / |  |  |  | / |  | / | / |  |  | / |  | / |
|  | *Apis cerana* | / |  | / | / | / |  | / | / |  | / |  |  | / | / | / |
|  | *Apis dorsata* |  |  |  | / |  |  |  |  |  |  |  |  |  |  |  |
|  | *Apis florea* |  |  |  |  | / |  |  |  |  |  |  |  |  | / |  |
|  | *Ceratina* | / |  | / |  | / |  | / | / |  |  |  |  | / |  |  |
|  | Formicidae |  |  |  | / |  |  | / | / |  | / | / |  | / | / | / |
|  | Hymenoptera (unidentified) | / | / | / | / | / |  |  |  | / | / |  |  |  | / | / |
|  | *Lasioglossum* | / | / | / | / | / | / | / | / | / |  | / |  | / | / | / |
|  | *Lipotriches* |  | / |  |  |  |  |  |  |  |  |  |  |  |  |  |
|  | *Megachile* | / |  |  |  |  |  |  |  |  |  |  |  |  |  |  |
|  | Meliponini | / | / | / |  |  |  | / | / |  |  |  |  | / | / |  |
|  | *Xylocopa aestuans* |  |  |  |  |  |  |  | / | / | / |  |  | / |  |  |
|  | *Xylocopa* | / |  |  |  |  |  |  |  |  |  |  |  |  |  |  |
| Lepidoptera | *Eurema* |  |  |  |  |  |  |  |  |  |  |  |  | / | / |  |
|  | Hesperiidae | / |  |  | / |  |  | / | / |  | / | / |  | / | / |  |
|  | *Hypolimnas* |  |  |  |  |  |  | / |  |  |  |  |  |  |  |  |
|  | *Junonia atlites* |  |  |  |  |  |  |  |  |  |  |  |  | / |  |  |
|  | *Junonia hierta* |  |  |  |  |  |  | / |  |  |  |  |  |  |  |  |
|  | *Junonia lemonias* |  |  |  |  |  |  |  |  |  | / |  |  | / |  |  |
|  | Lycaenidae |  |  | / |  | / |  |  |  |  | / |  |  | / | / |  |
|  | *Macroglossum* |  |  |  |  |  |  | / |  |  | / |  | / | / |  |  |
|  | *Matapa* |  |  |  |  |  |  |  |  |  |  |  | / |  |  |  |
|  | Noctuoidea |  |  |  |  |  |  |  |  |  | / |  |  |  |  |  |
|  | *Papilio demoleus* | / |  |  |  |  |  |  |  |  |  |  |  |  |  |  |
|  | Papilionoidea |  |  |  |  |  |  |  |  |  | / |  |  |  | / |  |
|  | *Parantica* |  |  |  |  |  |  |  |  | / |  |  |  |  |  |  |
|  | *Pareronia* |  |  |  |  |  |  |  |  |  | / |  |  |  |  |  |
|  | Pieridae |  |  |  |  |  |  |  |  |  | / |  |  | / |  |  |
| Orthoptera | Orthoptera (unidentified) |  |  |  |  |  |  | / |  |  |  |  |  |  |  |  |
| Unknown | Unknown |  |  |  | / | / |  |  |  |  |  |  |  |  | / |  |

Supplementary Table 4. The number of arthropod taxa observed and the comparative visitation rates (visits/hour; mean and standard error) at each of the 15 species of Convolvulaceae observed in Nong Khai, Thailand.

| **Plant Species** | **Visitors** | | | **Pollinators** | | |
| --- | --- | --- | --- | --- | --- | --- |
|  | **# Taxa** | **Total Visitation Rate** | | **# Taxa** | **Total Visitation Rate** | |
|  |  | **Mean** | **SE** |  | **Mean** | **SE** |
| *Argyreia capitiformis* | 18 | 7.93 | 3.63 | 10 | 4.99 | 3.16 |
| *Argyreia lanceolata* | 7 | 2.08 | 1.32 | 5 | 1.81 | 1.24 |
| *Camonea pilosa* | 17 | 31.91 | 8.93 | 8 | 20.29 | 5.74 |
| *Camonea vitifolia* | 14 | 7.72 | 3.05 | 8 | 6.23 | 2.82 |
| *Evolvulus nummularius* | 11 | 28.99 | 13.66 | 9 | 27.32 | 13.44 |
| *Hewittia malabarica* | 5 | 3.66 | 2.10 | 1 | 1.31 | 1.31 |
| *Ipomoea aquatica* | 24 | 12.59 | 2.95 | 13 | 3.39 | 0.91 |
| *Ipomoea batatas* | 12 | 35.57 | 2.65 | 8 | 17.51 | 2.57 |
| *Ipomoea cairica* | 15 | 8.70 | 3.95 | 7 | 2.01 | 0.95 |
| *Ipomoea nil* | 18 | 3.76 | 0.77 | 15 | 2.27 | 0.61 |
| *Ipomoea obscura* | 7 | 8.03 | 2.14 | 5 | 6.39 | 1.69 |
| *Ipomoea pileata* | 5 | 1.09 | 0.29 | 3 | 0.67 | 0.20 |
| *Merremia gemella* | 21 | 43.12 | 9.28 | 16 | 32.31 | 9.84 |
| *Merremia hederacea* | 15 | 47.10 | 17.20 | 15 | 40.98 | 15.84 |
| *Operculina turpethum* | 7 | 15.93 | 6.20 | 5 | 14.58 | 5.88 |
